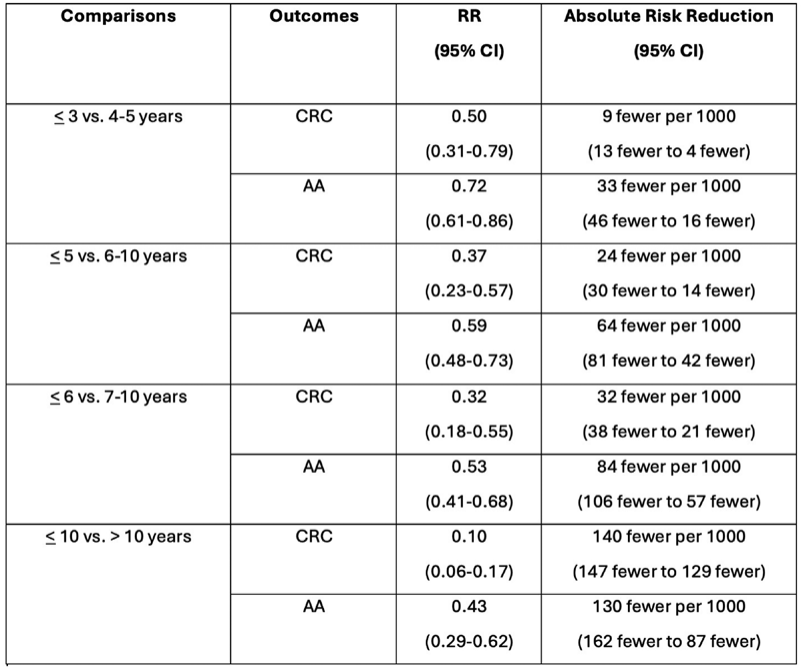# Poster Session I - A101 HOW SOON IS NECESSARY? A SYSTEMATIC REVIEW & META-ANALYSIS OF SURVEILLANCE INTERVALS AFTER RESECTION OF ≥ 3 NON-ADVANCED ADENOMAS

**DOI:** 10.1093/jcag/gwaf042.101

**Published:** 2026-02-13

**Authors:** H AlAwadhi, N Chang, G Leontiadis, N Forbes, Y Yuan, F Tse

**Affiliations:** McMaster University, Hamilton, ON, Canada; Gastroenterology, McMaster University, Hamilton, ON, Canada; McMaster University, Hamilton, ON, Canada; University of Calgary, Calgary, AB, Canada; McMaster University, Hamilton, ON, Canada; McMaster University, Hamilton, ON, Canada

## Abstract

**Background:**

Extending surveillance intervals for patients with > 3 small non-advanced adenomas (NAAs) is increasingly recommended, yet this trend is supported by systematic reviews using inappropriate comparator groups (polyp-free patients, general population) rather than comparisons of different intervals.

**Aims:**

This first systematic review and meta-analysis (SRMA) compares surveillance intervals in adults with a baseline finding of > 3 small NAAs.

**Methods:**

This SRMA was conducted to inform the CAG post-polypectomy surveillance guideline. We searched MEDLINE, EMBASE, and Cochrane Central Register of Controlled Trials up to March 2025 for randomized controlled trials (RCTs) and cohort studies comparing predefined surveillance intervals (<3 vs. 4–5 years, <5 vs. 6–10 years, <6 vs. 7–10 years, <10 vs. >10 years). Two reviewers independently performed study selection, data extraction, and quality assessment. Primary outcomes were colorectal cancer (CRC) incidence and mortality. Secondary outcomes included CRC stage, adverse events, all-cause mortality, and advanced adenoma (AA) incidence. Pooled risk ratios (RR) with 95% confidence intervals (CI) were calculated with a random-effects model. Heterogeneity was assessed by Chi^2^ (P < 0.15) and I^2^ tests (>25%). We assessed the certainty of evidence (CoE) with the GRADE approach.

**Results:**

From 3086 citations, 4 cohort studies (3 retrospective, 1 prospective) were included. No RCTs were identified. A dose-response relationship was observed: shorter surveillance intervals were associated with lower risks of CRC and AA across all comparisons (Table 1). The CoE for all outcomes was very low due to serious risk of bias and serious indirectness.

**Conclusions:**

Shorter surveillance intervals significantly reduce CRC and AA risks in patients with ≥3 small NAAs, supporting intensive surveillance and challenging prolonged intervals. Future research should identify higher-risk subgroups for personalized strategies.

**Funding Agencies:**

None